# 在线固相萃取-超高效液相色谱-串联质谱法测定水源水和饮用水中51种全氟和多氟烷基物质

**DOI:** 10.3724/SP.J.1123.2024.12020

**Published:** 2025-11-08

**Authors:** Yongyan CHEN, Jia LYU, Bixiong YE, Lan ZHANG, Yuanyuan WANG, Ning JIN

**Affiliations:** 中国疾病预防控制中心环境与人群健康重点实验室，中国疾病预防控制中心环境与 健康相关产品安全所，北京 100050; China CDC Key Laboratory of Environment and Population Health，National Institute of Environmental Health，Chinese Center for Disease Control and Prevention，Beijing 100050，China

**Keywords:** 在线固相萃取, 超高效液相色谱-串联质谱法, 全氟和多氟烷基物质, 水源水, 饮用水, online solid-phase extraction （online SPE）, ultra performance liquid chromatography-tandem mass spectrometry （UPLC-MS/MS）, per- and polyfluoroalkyl substances （PFASs）, raw water, drinking water

## Abstract

本研究建立了在线固相萃取-超高效液相色谱-串联质谱法快速筛查和检测水源水及饮用水中51种全氟和多氟烷基物质（PFASs）的分析方法。样品中加入甲酸铵及24种PFASs内标物质，混匀后使样品中甲酸铵浓度为2 mmol/L，PFASs内标物质质量浓度为2.5~50 ng/L，样品经0.22 μm孔径醋酸纤维素滤膜过滤后5 mL进样，经HLB在线固相萃取柱吸附后用2 mmol/L甲酸铵淋洗，以乙腈和2 mmol/L甲酸铵水溶液为流动相在线固相萃取后经BEH C_18_色谱柱进行分离，采用电喷雾离子源负离子模式电离、多反应监测模式检测，内标法定量。以水源水及饮用水作为基质，对其准确度和精密度进行方法学验证，结果表明：51种PFASs在其相应范围内线性关系良好（相关系数*r* ^2^>0.995），方法的检出限和定量限分别为0.03~1.5 ng/L和0.1~5.0 ng/L。将目标分析物在1、10、50 ng/L水平下加标，水源水和饮用水的加标回收率分别为60.2%~126.9%和60.4%~122.6%，相对标准偏差分别为0.3%~17.9%（*n*=6）和0.4%~17.7%（*n*=6）。用该方法测定水源水和饮用水中PFASs残留，结果显示，全氟烷基羧酸、全氟烷基磺酸、全氟烷基醚酸有较高水平的检出，其中水源水中检出质量浓度为0.1~209.7 ng/L，饮用水中检出质量浓度为0.1~63.6 ng/L。与离线固相萃取方法相比，本方法样品用量少，提高了样品采集便捷性的同时减少了内标物质用量。分析速度快且灵敏度高，重复性好，样品从在线富集至检测完成仅耗时20 min即可完成51种PFASs ng/L水平的同时测定。该方法适用于水源水和饮用水中全氟烷基羧酸、全氟烷基磺酸、全氟烷基醚酸、氟调聚物、氟烷基磺酰胺等多类PFASs的痕量测定，有效提高了水体中PFASs类物质的检测效率，实际应用价值较高。

## Abstract (en)

In this study， an online solid-phase extraction-ultra performance liquid chromatography-tandem mass spectrometry （online SPE-UPLC-MS/MS） method was established to rapidly screen and determine 51 per- and polyfluoroalkyl substances （PFASs） in raw and drinking water. Ammonium formate and 24 PFAS internal standards were added to each sample. The ammonium formate concentration in the sample was 2 mmol/L after mixing， and the contents of the PFAS internal standards ranged between 2.5 and 50 ng/L. Each sample was filtered through a 0.22-μm cellulose acetate filter， and a 5-mL aliquot was injected and adsorbed using an HLB online solid-phase extraction column and rinsed with 2 mmol/L ammonium formate. Acetonitrile and 2 mmol/L aqueous ammonium formate were used as mobile phases. Separation was performed on a BEH C_18_ chromatographic column with gradient elution. Electrospray ionization source negative ion mode and multiple reaction monitoring mode were used for detection， with quantification performed using the internal standard method. The method was validated by determining accuracies and precisions for the 51 PFASs using raw water and drinking water as matrices. Excellent linear relationships within their respective ranges were observed， with correlation coefficients （*r* ^2^）>0.995. The method exhibited limits of detection （LODs， *S/N*=3） and quantification （LOQs， *S/N*=10） of 0.03–1.5 and 0.1–5.0 ng/L， respectively. The PFASs were spiked at levels of 1， 10 and 50 ng/L， with spiked recoveries of 60.2%–126.9% and 60.4%–122.6% obtained for raw- and drinking-water samples， respectively， along with corresponding relative standard deviations （RSDs， *n*=6） of 0.3%-17.9% and 0.4%-17.7%， respectively. The developed method was used to determine PFAS residues in raw and drinking water， with perfluoroalkyl carboxylic acids， perfluoroalkyl sulfonic acids， and perfluoroalkyl ether acids detected at relatively high rates with contents of 0.1–209.7 and 0.1–63.6 ng/L in raw water and drinking water， respectively. Compared to offline solid-phase extraction methods， the developed method requires fewer samples， is more convenient from a sample-collection perspective， and uses lower quantities of internal standards. It also analyzes rapidly and is highly sensitive and reproducible. Only 20 min was required to determine the 51 PFASs at the ng/L level， from online enrichment to detection. The developed method is suitable for the trace determination of various types of PFAS， such as perfluoroalkyl carboxylic acids， perfluoroalkyl sulfonic acids， perfluoroalkyl ether acids， fluorotelomers， and fluoroalkyl sulfonamides， in raw and drinking water， thereby effectively boosting the detection efficiency for perfluoroalkyl substances in water in a manner that has substantial practical applications value.

全氟和多氟烷基物质（per- and polyfluoroalkyl substances，PFASs）作为新污染物已被我国纳入《重点管控新污染物清单（2023）》，已有研究表明PFASs具有肝脏毒性、生殖发育毒性和免疫毒性等，对环境及人类健康具有潜在危害。国内外环境水体中PFASs已有普遍检出^［1，2］^，受水处理工艺限制，饮用水中PFASs去除有限^［3］^，通过饮水暴露被确定为人类接触PFASs的主要途径，因此控制饮用水中PFASs污染至关重要。2023年4月1日起实施的《生活饮用水卫生标准》（GB 5749-2022）增加了对全氟辛酸（80 ng/L）和全氟辛烷磺酸（40 ng/L）的限值要求，2024年4月10日，美国环保总署颁布《国家一级饮用水法规》，其中对全氟辛酸、全氟辛烷磺酸、全氟己烷磺酸、全氟壬酸、六氟环氧丙烷二聚体酸等进行了限值要求，全氟辛酸和全氟辛烷磺酸限值低达4 ng/L。随着饮用水中PFASs的限值要求越来越严格，以及毒理学研究的不断深入，PFASs的限制种类数也在不断增加，这为水体中PFASs检测带来了新的挑战。

目前国内外水体中全氟及多氟烷基物质的相关前处理方法主要采用固相萃取法^［4-7］^，其耗时往往长于仪器分析检测时间，制约检测效率。本研究采用在线固相萃取技术，水样体积由几百毫升降低至几毫升，减少了内标物质使用量的同时提高了样品采集、储存、运输的便捷性；自动化程度高，无需氮吹、复溶等实验步骤，减少了离心管等实验器皿等带来的吸附或背景干扰。本方法分析速度快且灵敏度高，样品从在线富集至检测完成仅耗时20 min，且可完成ng/L水平的测定。建立的检测方法与已有方法^［8，9］^相比，除包含全氟烷基羧酸（perfluoroalkyl carboxylic acids，PFCAs）、全氟烷基磺酸（perfluoroalkane sulfonic acids，PFSAs）外，还包含PFCAs和PFSAs的替代物和前体物氟调聚物（fluorotelomers，FTs）、氟烷基磺酰胺（perfluoroalkyl sulfonamides，FASAs）及其替代物和前体物、全氟烷基醚酸（perfluoroalkylether carboxylic acids，PFECAs）、氯代多氟醚磺酸（chlorinated polyfluorinated ether sulfonates，Cl-PFESAs）等，涵盖了六氟环氧丙烷二聚酸（GenX）、9-氯-3-氧杂全氟壬烷磺酸（F-53B）、全氟壬烯氧基苯磺酸钠（OBS）等新型替代物，为水源水及饮用水中典型PFASs类污染物风险监测和健康风险评估提供技术支持。

## 1 实验部分

### 1.1 仪器、试剂与材料

ACQUITY UPLC-XEVO Micro TQS超高效液相色谱-串联质谱、OA system全自动在线前处理系统、在线固相萃取柱（HLB，30 mm×2.1 mm，20 μm）（美国Waters公司）；乙腈、甲醇（MS级，德国 Merck公司）；甲酸铵（MS级，美国Fisher公司）；0.22 μm醋酸纤维素滤膜（美国Pall公司）；51种PFASs标准品（100 μg/mL，溶剂为甲醇或乙腈，天津阿尔塔科技有限公司），24种同位素内标混合溶液（250~5 000 μg/L，加拿大Wellington公司）。

### 1.2 溶液的配制

混合标准储备液：分别准确移取1 000 µL 51种质量浓度为100 µg/mL的PFASs单标溶液，置于100 mL聚丙烯容量瓶中，用甲醇定容后混匀，-18 ℃避光保存备用。

混合标准使用液：准确移取100 µL 1 000 µg/L的PFASs混合标准储备液至10 mL聚丙烯容量瓶中，用甲醇定容后混匀，即为质量浓度为10 µg/L混合标准中间液；取2 mL混合标准中间液至100 mL聚丙烯容量瓶中，用2 mmol/L甲酸铵水溶液定容后混匀，现用现配。

同位素内标使用液：取100 μL同位素内标混合溶液至10 mL聚丙烯容量瓶中，用甲醇定容后混匀，即为质量浓度为2.5~50 μg/L的24种同位素内标混合使用液。

标准工作溶液配制：分别移取适量体积的200 ng/L混合标准使用液至50 mL聚丙烯容量瓶中，同时添加50 μL同位素内标混合使用液，以2 mmol/L甲酸铵水溶液为溶剂，配制质量浓度为0.1、0.2、0.5、1.0、2.0、5.0、20、50、100 ng/L的PFASs标准工作溶液，其中含24种同位素内标，其质量浓度为2.5~50 ng/L。

### 1.3 样品采集及预处理

使用棕色螺口聚丙烯瓶进行样品采集，满瓶采样，4 ℃冷藏避光保存。分析前准确量取20 mL样品，加入20 μL 2 mol/L的甲酸铵溶液，同时加入20 μL同位素内标混合使用液，使样品中含甲酸铵2 mmol/L。使用0.22 μm孔径醋酸纤维素滤膜对样品过滤后上机测定，进样体积为5 mL。

### 1.4 仪器条件

在线SPE：流动相Ａ为2 mmol/L甲酸铵水溶液；流动相Ｂ为甲醇；在线固相萃取柱为HLB（30 mm×2.1 mm，20 μm）；进样量为5 mL。梯度洗脱程序见表１，其中3.5~4.5 min为阀切换时间。

**表 1:** 液相色谱和在线固相萃取梯度洗脱条件

Liquid chromatography					Online-SPE				
Time/min	Flow rate/（mL/min）	*φ*（2 mmol/L ammonium formate）/%	*φ*（Acetonitrile）/%	Curve parameter	Time/min	Flow rate/（mL/min）	*φ*（2 mmol/L ammonium formate）/%	*φ*（Methanol）/%	Curve parameter
0	0.35	90	10	11^*^	0	2.00	100	0	11
3.5	0.01	90	10	11	0.5	1.00	100	0	11
4.5	0.35	90	10	6^*^	3.5	0.01	100	0	11
5.0	0.35	90	10	6	4.5	2.00	0	100	11
7.5	0.35	50	50	6	7.5	2.00	100	0	11
12.0	0.35	5	95	6	20.0	2.00	100	0	11
16.0	0.35	5	95	6					
16.5	0.35	90	10	6					
20.0	0.35	90	10	6					

6^*^： linear； 11^*^： keep the starting proportion and flow rate unchanged before the next step.

液相色谱分离：色谱柱（ACQUITY UPLC BEH C_18_，100 mm×2.1 mm，1.7 μm，美国Waters公司）；流动相A为2 mmol/L甲酸铵水溶液；流动相B为乙腈；柱温40 ℃。

电喷雾离子源负离子模式扫描，数据采集方式为多反应监测（MRM）；去溶剂气：高纯氮气，去溶剂温度500 ℃，流量900 L/h。锥孔气：高纯氮气，流量50 L/h。离子源温度150 ℃，碰撞气为氩气，毛细管电压0.5 kV。51种PFASs的离子对及质谱采集参数见表2。

**表 2:** 51种PFASs及24种内标的质谱参数

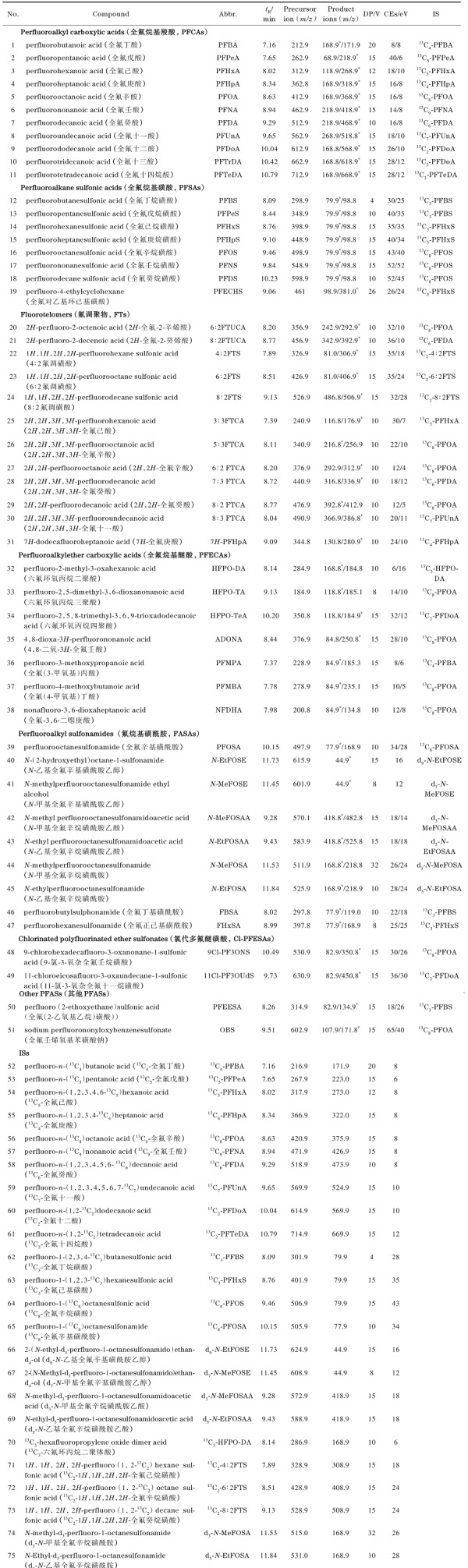

CE： collision energy； DP： declustering potential； * quantitative ion．

## 2 结果与讨论

### 2.1 色谱-质谱条件考察

本研究以全氟烷基羧酸、全氟烷基磺酸、氟调聚物、氟烷基磺酰胺、全氟烷基醚酸为主，碳链长度差异及不同的取代基团使各PFASs化学性质差异较大。质谱采集均采用负离子模式，全氟烷基羧酸、全氟烷基磺酸均采用脱氢负离子模式，［M-H］^-^为前体离子；部分全氟烷基醚酸发生源内裂解，其中六氟环氧丙烷二聚酸（HFPO-DA）、六氟环氧丙烷三聚酸（HFPO-TA）、六氟环氧丙烷四聚酸（HFPO-TeA）、全氟-3，6-二噁庚酸（NFDHA）源内裂解后分别以信号强度最高的［C_5_F_11_O］^-^、［C_3_F_7_O］^-^、［C_6_F_13_O_2_］^-^、［C_3_F_7_O_2_］^-^碎片为前体离子；*N*-甲基全氟辛基磺酰胺乙醇（*N*-MeFOSE）、*N*-乙基全氟辛基磺酰胺乙醇（*N*-EtFOSE）与流动相中甲酸根加合形成［M+HCOO］^-^，因此以［M+HCOO］^-^为前体离子，甲酸为产物离子。各PFASs特征离子对见表2。

超高效液相色谱系统流动相的梯度洗脱要与在线固相萃取柱的洗脱相协调。本研究选取乙腈和2 mmol/L甲酸铵水溶液为流动相，完成了PFASs的在线固相萃取洗脱色谱分离。流动相中添加甲酸铵促进了PFASs的离子化，提高了质谱响应值。51种PFASs（50 ng/L）混合标准溶液的总离子流色谱图见图1。

**图1:**
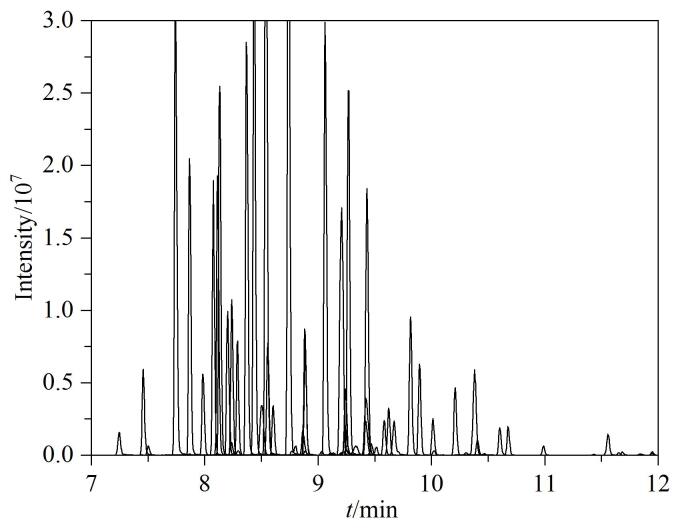
51种PFASs（50 ng/L）混合标准溶液的总离子流图

### 2.2 在线固相萃取条件的优化

根据已有报道在线固相萃取的检测方法中多选用WAX固相萃取柱富集PFASs ^［8，9］^。WAX固相萃取柱中为弱阴离子交换和水可浸润性的反相吸附剂填料，该萃取柱样品富集后需要使用碱性有机试剂洗脱。具有亲水亲脂平衡性的HLB固相萃取柱同样可实现对水体、土壤、食品等PFASs的富集^［10，11］^，但在线固相萃取方法中使用较少。本研究重点考察了HLB在线固相萃取柱对PFASs的富集效果，同时对比了HLB和X Bridge C_18_通用型反相吸附固相萃取柱。实验结果显示，全氟丁酸和全氟（3-甲氧基）丙酸在PFASs中碳链较短，亲水性强，在X Bridge C_18_柱上保留弱于HLB柱，回收率较低，因此本研究选取HLB固相萃取柱。

### 2.3 样品采集及预处理条件优化

#### 2.3.1 进样溶剂优化

实验对比了样品中添加不同浓度（0~20 mmol/L）甲酸铵对PFASs分析的影响，使用外标法定量，对比不同浓度的甲酸铵体系下样品加标与同体系下标准工作溶液的响应值，计算回收率。如图2所示，综合PFASs响应值及回收率，表明样品在2 mmol/L甲酸铵体系下上样至固相萃取柱，并使用2 mmol/L甲酸铵水溶液淋洗效果最佳。同时实验对比了样品中含不同体积分数（0、5%、10%、20%）甲醇对检测响应值的影响，结果显示样品中添加甲醇后，碳链较短、*M*_r_为200~300的PFASs响应值有不同程度的降低，可能甲醇降低了固相萃取柱对短链和更亲水的PFASs的吸附率，导致短链和更亲水的PFASs损失，其中，全氟丁酸（PFBA）、全氟戊酸（PFPeA）、全氟（3-甲氧基）丙酸（PFMPA）、全氟（4-甲氧基）丁酸（PFMBA）、2*H*，2*H*，3*H*，3*H*-全氟己酸（3∶3FTCA）最为明显，当样品中甲醇体积分数为20%时，响应值仅为样品中不含甲醇时的20%~40%。但对于长碳链PFASs，随着碳链长度增加，甲醇体积分数越大，响应值增强，这与文献报道的结果相一致^［5］^，考虑到短链PFASs的萃取效率以及甲醇的加入会稀释样品中PFASs的浓度，因此在样品中不添加甲醇。

**图2:**
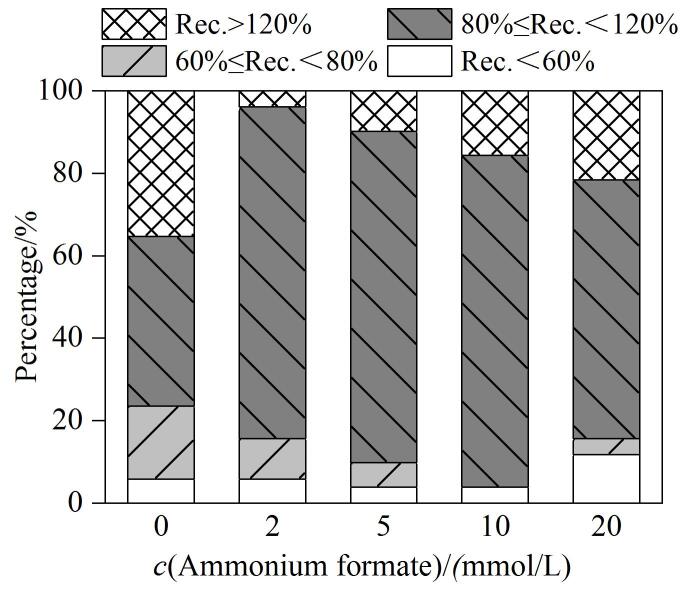
甲酸铵的浓度对回收率的影响

#### 2.3.2 样品稳定性

PFASs为持久性有机污染物，已有报道显示全氟烷基羧酸及全氟烷基磺酸在水中稳定性低于在甲醇等有机试剂中^［12］^。本研究进行了样品稳定性试验，在水样中分别加入低、中、高3个水平（10、50、100 ng/L）的PFASs标准混合溶液，混匀后将各水平加标样品分装至聚丙烯采样瓶中，分别在25 ℃和4 ℃条件下储存，在1、3、5、7天分别测定各PFASs的峰面积，与新配制的PFASs的峰面积进行对比，计算不同储存温度和储存时间下的平均回收率。实验结果显示，除氟调羧酸（fluorotelomer carboxylic acid， FTCAs）和氟调不饱和羧酸（fluorotelomer unsaturated carboxylic acid，FTUCAs）外，本研究中其他PFASs在冷藏或常温条件下储存后测定响应值均未显著变化。FTCAs和FTUCAs在常温放置会发生不同程度的降解，而低温冷藏条件下较为稳定，如图3所示。因此建议水样采集后要低温冷藏保存，并尽快测定。

**图3:**
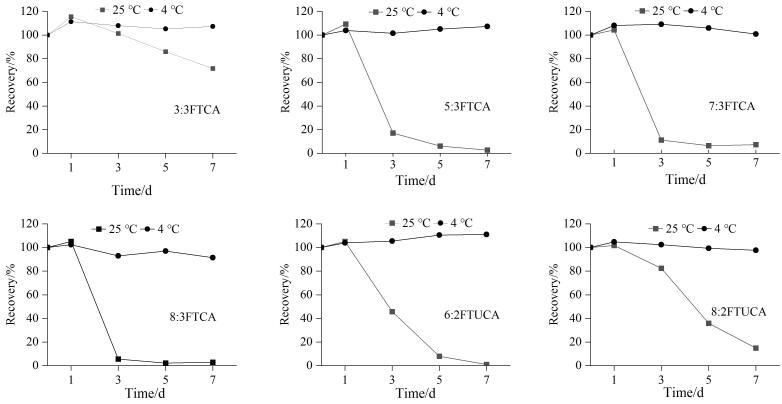
样品的保存温度和时间对回收率的影响

#### 2.3.3 滤膜吸附的影响

在线固相萃取系统一般进样体积为2~10 mL，为防止水样中泥砂等微小固体颗粒物质堵塞分析柱，除了在分析柱前安装在线过滤器外，样品上样前经0.22 μm滤膜过滤尤为重要。水样过滤常用的亲水性聚四氟乙烯（polytetrafluoroethylene，PTFE）及聚偏二氟乙烯（polyvinylidenefluoride，PVDF）含有PFASs，应避免使用，市售玻璃纤维滤膜孔径过大，无法满足对微小颗粒物的去除。因此本研究考察了聚醚砜（polyether sulfone，PESF）、混合纤维素酯（mixed cellulose ester，MCE）、醋酸纤维素（cellulose acetate，CA）、再生纤维素（regenerated cellulose，RC）4种材质滤膜对PFASs的吸附，将含有20 ng/L PFASs的加标样品经不同种类的滤膜过滤，滤液弃去前5 mL后收集20 mL滤液进样测定，以外标法定量，通过回收率考察滤膜对PFASs的吸附性。如图4所示，醋酸纤维素滤膜对PFASs吸附效应最弱，51种PFASs中，回收率大于60%的有42种。本研究中回收率小于60%的PFASs为长链PFASs，*M*_r_为500以上，主要包含长链全氟羧酸，如全氟十三酸、全氟十四烷酸，以及氟烷基磺酰胺类，如*N*-甲基全氟辛烷磺酰胺、*N*-乙基全氟辛烷磺酰胺等。PFASs碳链偏长极性增强，导致水系滤膜过滤会有相对较强的吸附，可通过添加该物质的同位素内标进行校准以提高PFASs测定的准确性。

**图4:**
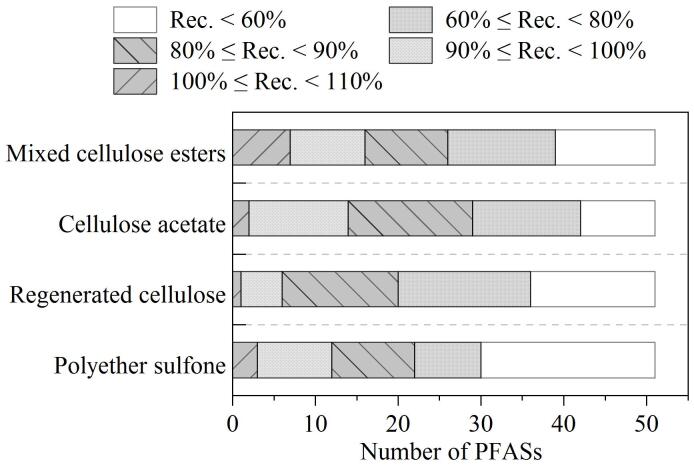
不同材质滤膜对PFASs回收率的影响

### 2.4 方法学考察

#### 2.4.1 线性关系和检出限

按照1.2节描述配制不同质量浓度的PFASs标准工作溶液，标准工作溶液经在线固相萃取处理后进行测定，以各PFASs的质量浓度为横坐标，以各组分和对应内标的峰面积比值为纵坐标进行线性回归分析。实验结果表明，51种PFASs在各自的范围内线性关系良好，线性相关系数（*r* ^2^）>0.995。以3倍和10倍信噪比（*S/N*）对应的质量浓度确定本方法的检出限（LOD，*S/N*=3）和定量限（LOQ，*S/N*=10），如表3所示，51种PFASs的检出限为0.03~1.5 ng/L，定量限为0.1~5.0 ng/L。美国《国家一级饮用水法规》中PFASs有限值要求的指标（PFOA、PFOS、PFHxS、PFNA、HFPO-DA）均在本研究方法内，且定量限均小于限值^［13］^。

**表3:** 51种PFASs的线性范围、线性方程、相关系数、检出限和定量限

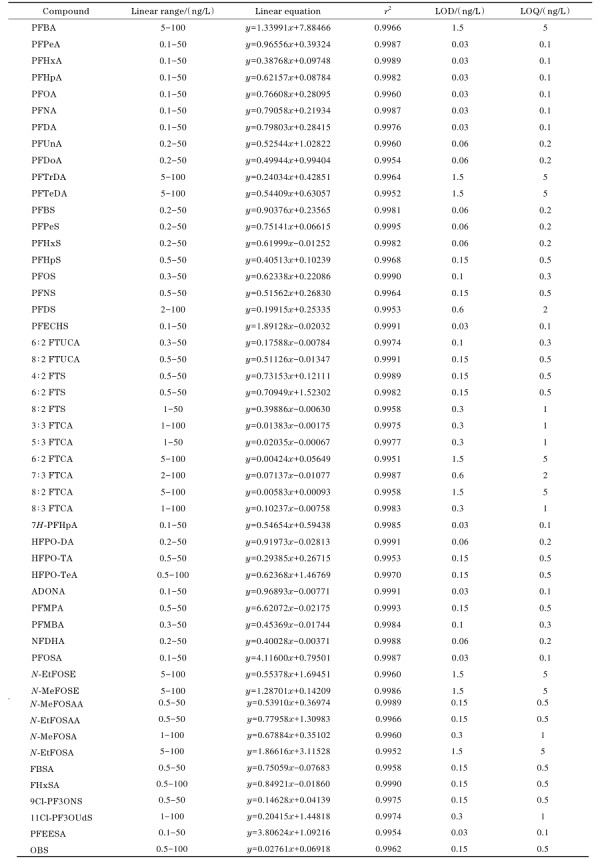

*y*： peak area ratio of PFASs to internal standard； *x*： mass concentration， ng/L.

#### 2.4.2 回收率和精密度

分别向空白饮用水及水源水中添加低、中、高（1、10、50 ng/L）3个水平的混合标准溶液进行加标回收试验，每个水平平行进行6组，计算回收率及相对标准偏差。如表4所示，饮用水中51种PFASs在3个水平下的加标回收率（*n*=6）分别为62.6%~124.2%、62.1%~117.9%、60.4%~122.6%，相对标准偏差分别为1.7%~17.7%、0.4%~13.6%、0.5%~12.4%。水源水中51种PFASs在3个水平下的加标回收率（*n*=6）分别为68.5%~119.1%、60.2%~126.9%、63.1%~115.7%，相对标准偏差分别为1.1%~17.9%、0.5%~16.1%、0.3%~13.7%。

**表4:** 51种PFASs的加标回收率和相对标准偏差（*n*=6）

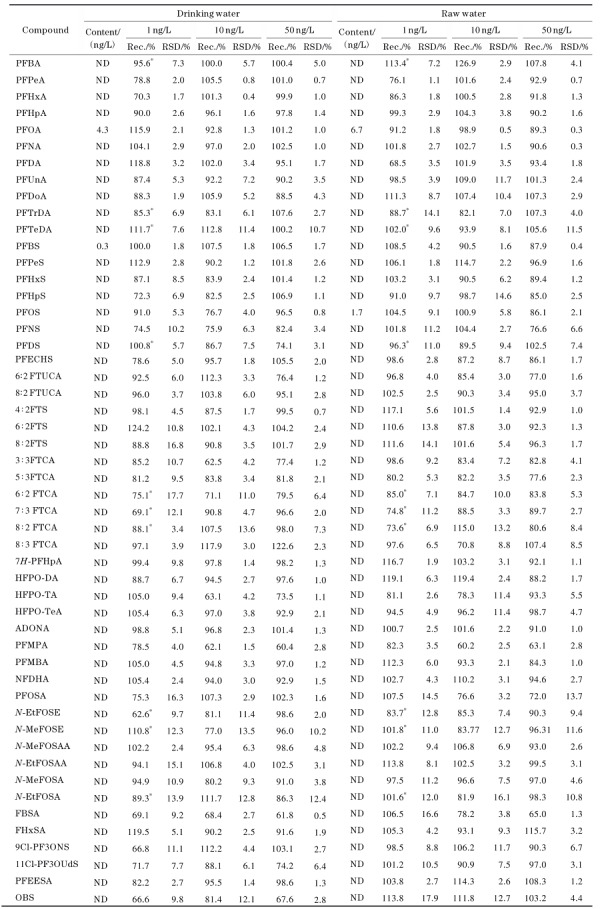

* Spiked 5 ng/L； ND： not detected.

### 2.5 实际样品的测定

用本方法分别对我国中部及东部地区12个城市的水源水及饮用水进行51种PFASs的测定，其中水源水样品16件，饮用水样品16件。在水源水中检出25种PFASs，水平在0.1~209.7 ng/L；饮用水中检出18种，水平在0.1~63.6 ng/L，全氟羧酸、全氟磺酸、全氟烷基醚酸等有较高水平的检出，检出指标及含量范围见表5。本方法对水源水和饮用水中PFASs检测具有适用性。

**表 5:** 水源水和饮用水中PFASs的检出情况

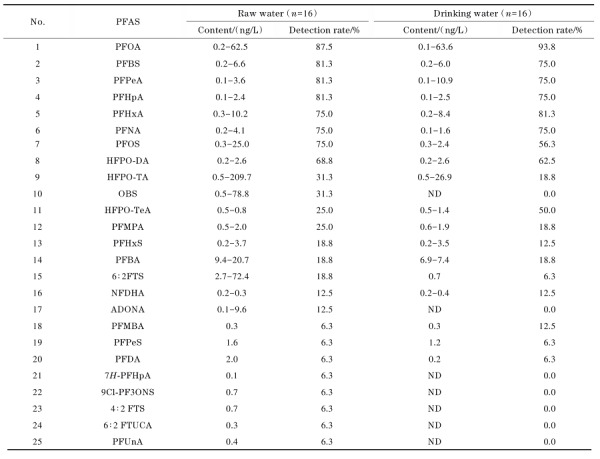

### 2.6 与其他方法对比

离线SPE前处理方法中上样、洗脱、氮吹、复溶等时间长、步骤多，在样品处理过程中室内环境、所用的实验器皿可能会污染样品，导致检测背景升高；相比之下，在线SPE自动化程度高，大大提高了检测效率，样品富集及分析时长仅20 min。样品在处理过程中，为校准目标分析物损失或样品基质干扰，通常会添加同位素内标进行定量。离线固相萃取所需样品量为250~1 000 mL，内标物质用量大，而在线固相萃取样品量小，大大降低了内标物质的使用量。本方法适用的51种PFASs包含了US EPA Method 537.1中18种PFASs 和US EPA Method 533中25种PFASs的全部测定，表 6列出了本法与现行的美国与中国标准方法的方法指标比较，虽然本方法采用的在线 SPE 样品用量少，但富集后全部洗脱至质谱检测，富集倍数与离线 SPE 相当，具有高灵敏度的优点，适合于水体中痕量PFASs的快速测定。

**表 6:** 本方法与其他方法的比较

Detection method	Samples	Number of detected PFASs	Type of detected PFASs	LOQs/（ng/L）	Sample volume/mL	Ref.
Online-SPE-LC-MS/MS	raw water， drinking water	51	PFCAs， PFSAs， PFECAs， FASAs， FTs， Cl-PFESAs， PFESAs	0.1-5	5	this study
SPE-LC-MS/MS	drinking water	18	PFCAs， PFSAs， PFECAs， FASAs， Cl-PFESAs	0.53-6.3	250	［^14^］
SPE-LC-MS/MS	drinking water	25	PFCAs， PFSAs， PFECAs， FTs， PFESAs	1.4-16	250	［^15^］
SPE-LC-MS/MS	drinking water	11	PFCAs， PFSAs	3-5	1000	［^16^］
SPE-LC-MS/MS	surface water， ground water， waste water， seawater	2	PFCAs， PFSAs	2.0-2.4	500	［^17^］

## 3 结论

本研究将在线固相萃取前处理系统与超高效液相色谱-串联质谱相结合，建立了快速筛查和定量水源水和饮用水中51种PFASs的方法，实现了对水体中PFCAs、PFSAs、PFECAs、FASAs、FTs等多类PFASs的同时测定，方法准确、灵敏，操作简单，有效提高了水体中PFASs的检测效率。同时本研究发现水体中FTCAs和FTUCAs在常温下易转化或降解，低温可增强其在水体中的稳定性，水样采集后应低温保存并尽快测定。采用所建立的方法对中部及东部地区部分水源水及出厂水进行分析，发现PFASs类污染已普遍存在，需要进一步加强对饮用水中PFASs类污染物监测和风险评估。
